# Repeated Sieving for Prediction Model Building with High-Dimensional Data

**DOI:** 10.3390/jpm14070769

**Published:** 2024-07-19

**Authors:** Lu Liu, Sin-Ho Jung

**Affiliations:** Department of Biostatistics and Bioinformatics, Duke University, Durham, NC 27708, USA; lu.liu1@duke.edu

**Keywords:** Cox regression, logistic regression, machine learning, ROC curve, variable selection

## Abstract

**Background**: The prediction of patients’ outcomes is a key component in personalized medicine. Oftentimes, a prediction model is developed using a large number of candidate predictors, called high-dimensional data, including genomic data, lab tests, electronic health records, etc. Variable selection, also called dimension reduction, is a critical step in developing a prediction model using high-dimensional data. **Methods**: In this paper, we compare the variable selection and prediction performance of popular machine learning (ML) methods with our proposed method. LASSO is a popular ML method that selects variables by imposing an $L_{1}$-norm penalty to the likelihood. By this approach, LASSO selects features based on the size of regression estimates, rather than their statistical significance. As a result, LASSO can miss significant features while it is known to over-select features. Elastic net (EN), another popular ML method, tends to select even more features than LASSO since it uses a combination of $L_{1}$- and $L_{2}$-norm penalties that is less strict than an $L_{1}$-norm penalty. Insignificant features included in a fitted prediction model act like white noises, so that the fitted model will lose prediction accuracy. Furthermore, for the future use of a fitted prediction model, we have to collect the data of all the features included in the model, which will cost a lot and possibly lower the accuracy of the data if the number of features is too many. Therefore, we propose an ML method, called repeated sieving, extending the standard regression methods with stepwise variable selection. By selecting features based on their statistical significance, it resolves the over-selection issue with high-dimensional data. **Results**: Through extensive numerical studies and real data examples, our results show that the repeated sieving method selects far fewer features than LASSO and EN, but has higher prediction accuracy than the existing ML methods. **Conclusions**: We conclude that our repeated sieving method performs well in both variable selection and prediction, and it saves the cost of future investigation on the selected factors.

## 1. Introduction

In personalized medicine, it is important to predict patients’ short-term or long-term outcomes from their past or current characteristics, called features, predictors, or covariates. Usually, the original clinical data have a large number of features that are possibly associated with an outcome. The features consist of various types of data from different sources, such as genomic experiments, health screening, wearable devices, lab tests, and so on. Oftentimes, the number of candidate features is larger than the number of cases, which makes the traditional statistical methods unusable for this kind of data analysis. So, in prediction model building, feature selection is a critical step to improve the performance of fitted prediction models. Machine learning (ML) methods have emerged as a strong tool for building prediction models with high-dimensional data [1]. While the number of candidate features is big, usually only a small number of them are truly associated with the outcome.

Least absolute shrinkage and selection operator (LASSO [2]) is one of the most popular ML methods due to its strong prediction accuracy using different types of outcome variables. However, it is well-known that LASSO tends to over-select features [3]. If a prediction model over-selects features, many of the features included in a fitted prediction model will be falsely selected. In this case, the falsely selected features will act like random errors in the fitted model, so that they are deleterious to the prediction accuracy. Selecting too many features can also incur excessive cost since those features should be continuously collected to predict the outcome of future subjects. For example, suppose that we want to develop a prediction model using gene expression data. In such a project, we usually start with commercial microarray chips covering thousands of genes to identify the genes that are believed to be associated with a patient’s outcome. Once a prediction model is developed and validated, it will be used to predict the outcome of future patients. To this end, we would develop customized chips that include only the selected genes to increase the accuracy of assay and cut down the price of arrays [4]. In this case, if the fitted model includes too many genes, the price of the customized chips will be high and the performance of the assay to measure the expression of included genes will be compromised. The long-term use of a fitted prediction model can be very costly if it includes features that are obtained from expensive or time-consuming procedures, especially if they are not really associated with the outcome.

By using an $L_{1}$-norm penalty, LASSO selects features based on the size of their regression estimates, rather than the statistical significance associated with the outcome. If all features have the same distribution, a variable selection based on the size of regression estimates will be identical to that based on statistical significance. As such, before applying an ML method, we standardize the features as an effort to make their distributions similar. No existing standardization method, however, makes the distributions of a large number of features perfectly identical. In particular, if different variable types (e.g., discrete and continuous variable types) are mixed among features, it will be impossible to unify their distributions.

Elastic net (EN [5]) uses a combination of $L_{1}$-norm and $L_{2}$-norm penalties, so that its regularization is even less strict in variable selection than the $L_{1}$-norm penalty for LASSO. Hence, EN has a more serious over-selection problem.

To resolve these issues of LASSO and EN, Liu et al. [3] proposed to use a standard (un-penalized) regression method combined with the stepwise forward variable selection procedure to develop prediction models with high-dimensional data. The ML methods provide 0-shrinkage estimators, even for a reduced model, while standard regression models with stepwise selection provide the maximum likelihood estimators (MLEs). Furthermore, contrary to LASSO and EN, the standard regression methods select features based on the statistical significance of the predictors. Liu et al. [3] show that standard regression models equipped with a stepwise variable selection method include much fewer predictors in the fitted prediction model while maintaining strong prediction accuracy—comparable to LASSO and EN. However, as the dimension of features increases, the computing of stepwise selection can be quite heavy since it goes through every combination of predictors until it finds a set of all significant predictors.

For prediction with a large number of candidate predictors, we propose a repeated sieving method that partitions the predictors into many small blocks, so that traditional regression with stepwise variable selection can be conducted quickly and efficiently. Often, predictors are correlated. In this case, if there are two correlated covariates that are associated with the outcome, then both of them should be included in the same block for unbiased estimation [6]. When there are true covariates that are correlated with each other, the chance that all of them belong to the same block will be very small, especially if the block size is small and the number of candidate features is large. To increase this chance, we permute the covariates and apply the same procedure repeatedly. The final set of candidate predictors will be the union of the covariates selected (or sieved) from each block and each permutation. We may conduct the procedure of Liu et al. [3] to fit a prediction model if the number of covariates in the final set is not too large, say, smaller than 1000. Otherwise, we may apply another round of repeated sieving to the reduced set of features before conducting the procedure of Liu et al. [3]. This approach divides a complicated and heavy-computing task into multiple simple and fast-computing tasks, which enhances the efficiency and accuracy of the model fitting. Note that repeated sieving can also make use of parallel computing, which will further expedite the computations.

There have been numerous papers comparing the performance of logistic regression and some ML methods [7,8,9,10,11,12] and some studies comparing the performance of ML methods for survival outcomes with that of Cox regression [13]. Some previous studies also compare the prediction power of stepwise variable selection with LASSO and EN [14,15]. Most of the studies above are anecdotal in the sense that their findings are based on real data analyses without any systematic simulation studies. Furthermore, their example data sets are not really high dimensional because the number of features is not very large while the number of cases is large. For this kind of data set, standard regression methods work perfectly and we do not need a ML method. In this sense, they do not really evaluate the performance of ML methods for high-dimensional data. Hastie et al. [16] conducted extensive numerical studies to compare the prediction accuracy of forward stepwise selection and LASSO using continuous outcomes, but the stepwise method they use selects the covariates based on model fitting criteria such as R-square or AIC/BIC instead of un-penalized MLE or *p*-values from hypothesis testing.

In this paper, we compare the variable selection performance and prediction accuracy of LASSO, EN, and our repeated sieving method. In our numerical studies, we conduct extensive simulations with binary outcomes using logistic regression and survival outcomes using Cox regression model, and demonstrate our findings using a real data example.

## 2. Materials and Methods

We want to compare the performance of our repeated sieving method with LASSO and EN using simulations and the analysis of real data. We introduce our proposed method and briefly review LASSO and EN, and introduce the measurements to evaluate the performance of fitted prediction models.

Our repeated sieving method is nested with a standard regression method and stepwise variable selection [3]. The standard regression method for binary outcome is logistic regression with stepwise variable selection (L-SVS), and that for time-to-event outcome is Cox regression with stepwise variable selection (C-SVS). Suppose that there are *n* subjects, and we observe an outcome variable *y* and *m* features $\left(x_{1},\ldots ,x_{m}\right)$ from each subject. The observed data set will look like $\left\{y_{i},\left(x_{1i},\ldots ,x_{mi}\right),i=1,\ldots ,n\right\}$. For high-dimensional data, *m* is much larger than *n*, while the number of features that are truly associated with the outcome, denoted as $m_{1}$, is often very small. We consider 50–50 hold-out to partition a given data set into training and validation sets.

### 2.1. Statistical Models

Let $Z={\left(z_{1},\ldots ,z_{k}\right)}^{T}$ denote a subset of features that are possibly associated with an outcome variable, and let $\beta ={\left(\beta_{1},\ldots ,\beta_{k}\right)}^{T}$ denote their regression coefficients. We assume that *k* is much smaller than the number of cases, *n*.

#### 2.1.1. Logistic Regression

Logistic regression is a popular method to associate a binary outcome variable *y* with covariates *Z* [17]. Suppose that, from *n* patients, we have data $\left\{\left(y_{i},Z_{i}\right),i=1,\ldots ,n\right\}$, where $y_{i}$ is a binary outcome that takes 1 if patient *i* has a response and 0 otherwise. A logistic model associates the response rate $P(y_{i}=1)$ with covariates by

$$\log\frac{P(y_{i}=1)}{1-P(y_{i}=1)}=\beta_{0}+\beta^{T}Z_{i}$$

where $\beta_{0}$ is an unknown intercept term.

Then, given covariates $Z_{i}$, outcomes $y_{i}$ are independent Bernoulli random variables with response rate

$$P\left(y_{i}=1\right)=\frac{\exp(\beta_{0}+\beta^{T}Z_{i})}{1+\exp(\beta_{0}+\beta^{T}Z_{i})}$$

so that regression estimates $\left({\hat{\beta }}_{0},{\hat{\beta }}_{\tilde{1}},\ldots ,{\hat{\beta }}_{\tilde{k}}\right)$ are obtained by maximizing the log-likelihood function

$${𝓁}_{1}\left(\beta_{0},\beta \right)=\sum_{i=1}^{n}\left[y_{i}\left(\beta_{0}+\beta^{T}Z_{i}\right)-\log\left\{1+\exp\left(\beta_{0}+\beta^{T}Z_{i}\right)\right\}\right]$$

with respect to $\left(\beta_{0},\beta_{1},\ldots ,\beta_{k}\right)$.

#### 2.1.2. Cox Proportional Hazards Model

Due to its efficiency and robustness, Cox’s [18] proportional hazards model is popularly used to relate a time-to-event endpoint with covariates. For subject i(=1,…,n), let $y_{i}$ denote the minimum of survival time and censoring time, and $\delta_{i}$ the event indicator that takes 1 if subject *i* has an event and 0 otherwise. The data from *n* patients are summarized as $\left\{\left(y_{i},\delta_{i}\right),Z_{i},i=1,\ldots ,n\right\}$. We assume that, conditioning on covariates, censoring time is independent of survival time. Using the Cox proportional hazards model, the hazard function, $h_{i}\left(t\right)$, of patient *i* is expressed as

$$h_{i}\left(t\right)=h_{0}\left(t\right)e^{\beta^{T}Z_{i}}$$

where $h_{0}\left(t\right)$ is an unknown baseline hazard function. The regression estimates $\left({\hat{\beta }}_{1},\ldots ,{\hat{\beta }}_{k}\right)$ are obtained by maximizing the partial log-likelihood function

$${𝓁}_{2}\left(\beta \right)=\sum_{i=1}^{n}\delta_{i}\left\{Z_{i}-\frac{\sum_{j=1}^{n}I\left(y_{j}\geq y_{i}\right)Z_{j}\exp\left(\beta^{T}Z_{j}\right)}{\sum_{j=1}^{n}I\left(y_{j}\geq y_{i}\right)\exp\left(\beta^{T}Z_{j}\right)}\right\}$$

where I(·) is the indicator function.

#### 2.1.3. Stepwise Variable Selection

Variable selection, also called dimension reduction, is an important procedure in building prediction models using high-dimensional data because the number of candidate features *m* is much larger than the sample size *n*, while the number of features that are truly associated with outcome is small. Popular variable selection methods for standard regression methods include forward stepwise procedure, backward elimination procedure, and all possible combination procedure. Backward elimination and all possible combination procedures are not workable for high-dimensional data because the estimation procedure of regression models does not converge for the models with a large number of features.

On the contrary, forward selection procedure is very useful, especially when the number of covariates that are truly related with the outcome is small. For a forward selection procedure, we specify two alpha values, $\alpha_{1}$ for insertion and $\alpha_{2}\left(\geq \alpha_{1}\right)$ for deletion. The selection procedure starts with an intercept term (or an empty model for regression models not including an intercept term, like Cox’s proportional hazards model). In each step, it selects the most significant covariate if its *p*-value is smaller than $\alpha_{1}$, and the extraneous covariates are removed if they become insignificant after adding a new variable (i.e., if their *p*-values are larger than $\alpha_{2}$). This procedure continues until no more variables are added to the current model. We can control the number of features to be selected for a prediction model by using appropriate $\alpha_{1}$ and $\alpha_{2}$ values.

Some data analysis computer programs, e.g., SAS, use penalized likelihood criteria for variable selection, such as Akaike information criterion or Bayesian information criterion, rather than performing hypothesis testing to calculate *p*-values. Using these methods, we do not know how significant the selected covariates are and cannot control the number of selected features.

### 2.2. Machine Learning Methods

#### 2.2.1. LASSO

LASSO is a regularized regression method, imposing an $L_{1}$-norm penalty to the objective function of traditional regression models. For a binary outcome, the negative log-likelihood function of a logistic regression is added with an $L_{1}$-norm penalty and the regression estimates are obtained by minimizing [2]

$$-{𝓁}_{1}\left(\beta_{0},\beta \right)+\lambda ||\beta_{0},\beta {\left|\right|}_{1}$$

with respect to $\left(\beta_{0},\beta ,\lambda \right)$.

For a time-to-event outcome, an $L_{1}$-norm penalty is imposed to the negative log-partial likelihood function of a proportional hazards model, and the regression estimates are obtained by minimizing [19]

$$-{𝓁}_{2}\left(\beta \right)+{\lambda ||\beta ||}_{1}$$

with respect to (β,λ). In this paper, the tuning parameter λ is determined by minimizing the regularized objective function through an internal cross-validation.

#### 2.2.2. Elastic Net

EN is a generalized regularized regression method, imposing a combination of $L_{1}$- and $L_{2}$-norm penalties to the objective function. For logistic regression for a binary outcome, the regression estimates are obtained by minimizing [5]

$$-{𝓁}_{1}\left(\beta_{0},\beta \right)+\lambda_{1}\left|\right|\beta_{0}{,\beta ||}_{1}+\lambda_{2}\left|\right|\beta_{0},\beta {\left|\right|}_{2}^{2}$$

with respect to $\left(\beta_{0},\beta ,\lambda_{1},\lambda_{2}\right)$.

For Cox regression for a time-to-event outcome, regression estimates are obtained by minimizing [5]

$$-{𝓁}_{2}\left(\beta \right)+\lambda_{1}{\left||\beta |\right|}_{1}+\lambda_{2}{\left||\beta |\right|}_{2}^{2}$$

with respect to $\left(\beta ,\lambda_{1},\lambda_{2}\right)$. The tuning parameters $\left(\lambda_{1},\lambda_{2}\right)$ are obtained by minimizing the regularized objective function using an internal cross-validation as in LASSO.

### 2.3. Repeated Sieving Method

In high-dimensional data, often the number of features that are truly associated with the outcome is small while the number of candidate features is very big. In this case, Liu et al. [3] have shown that, compared to LASSO and EN, traditional regression methods with stepwise variable selection (R-SVS) has similar or even better prediction performance with much fewer selections owing to their higher selection precision. In this paper, we consider higher-dimensional data with a level of features greater than 10K and possible dependency among features. To address the issue of heavy computation and selection error due to multi-collinearity, we propose a repeated sieving approach that conducts a two- or multi-step variable selection.

If the dimension, *m*, of a data set is really high, Liu et al.’s [3] R-SVS is very time-consuming since it progresses through every combination of each low dimensional set of features and conducts hypothesis testing on the selected features. Our repeated sieving method partitions the whole features into many small blocks, say, of size $m_{0}=50$, and sorts (or sieves) significant features from each block. Computing for R-SVS with small blocks will be very fast. The features selected from $m/m_{0}$ blocks are candidate features that are truly associated with the outcome.

Note that features belonging to different blocks are never included in a common joint regression model at this step. According to Gail et al. [6], if a covariate is not associated with the outcome or independent of other covariates that are included in the model, then a regression model with the covariate missing has no bias issue at all. However, if two covariates are correlated and both are truly associated with the outcome, then both covariates should be included in a regression model for unbiased estimation of the regression coefficients. In order to take care of this issue, in the next step, we randomly permute the features and partition them into $m/m_{0}$ blocks of size $m_{0}$, again with the hope that two dependent true features are assigned to the same block. We apply the same sieving procedure to the permuted data as that in the first step. Since the block size is very small, e.g., 50, compared to the dimension of the whole data *m*, we repeat the permutations many times, say, P=100 times. The features selected from the multiple permutations are candidate features to be selected for the final prediction model. Note that the set of selected features may grow as we repeat more permutations. If the set of selected features is not so big (for example, includes less than 1000 features), then we apply R-SVS to this set of features. If this set of selected features is still too big, however, we may apply another round of sieving to this reduced set of features.

In each stage of repeated sieving, we need to specify two α values, $\alpha_{1}$ for insertion and $\alpha_{2}$ for deletion, to be used in R-SVS for each block. In the variable selection for the final model, we may use different alpha values of $\alpha_{3}$ and $\alpha_{4}$ for deletion.

- For the original data set with *n* cases and *m* features, permute the order of features;
- For each permuted data, partition the features into $m/m_{0}$ blocks and apply R-SVS to each block using $\left(\alpha_{1},\alpha_{2}\right)$;
- Permute the data set *P* times in total, and the sieved candidate features are the union of the selections from all permutations;
- Apply R-SVS to the set of sieved features using $\left(\alpha_{3},\alpha_{4}\right)$ to select features for the final model.

### 2.4. Performance Measurements

The variable selection performance of these prediction methods are evaluated by the total number of selected covariates and the number of selected covariates that are truly associated with the outcome, where the proportion of true selection over total selection shows the selection precision. Let $r={\hat{\beta }}^{T}Z$ denote the risk score, where *Z* is the vector of features included in the fitted prediction model and $\hat{\beta }$ is the vector of their regression estimates. For a data set with a binary outcome, $\left\{\left(y_{i},Z_{i}\right),i=1,\ldots ,n\right\}$, the prediction performance of a fitted prediction model can be evaluated by the area under the curve (AUC) of the ROC curve generated using $\left\{\left(y_{i},r_{i}\right),i=1,\ldots ,n\right\}$, where $r_{i}={\hat{\beta }}^{T}Z_{i}$. An accurate prediction model has a large AUC value close to 1. On the other hand, for a data set with survival outcome, $\left\{\left(y_{i},\delta_{i}\right),Z_{i},i=1,\ldots ,n\right\}$, the prediction performance of a fitted prediction model can be evaluated by Harrell’s concordance C-index between $\left(y_{i},\delta_{i}\right)$ and $r_{i}$. We also compute $-\log_{10}\left(p\text{-}\mathrm{value}\right)$ of the univariate Cox proportional hazards model, regressing $\left(y_{i},\delta_{i}\right)$ on $r_{i}$. For a survival outcome, an accurate prediction model has a large C-index and a large negative log *p*-value.

All data analyses for model fitting are conducted using open-source R software, R Foundation for Statistical Computing, under version 3.6.0. The R packages and functions we use for LASSO and EN is cv.glmnet from glmnet package. We developed our own R function for R-SVS for repeated sieving based on specified alpha values for insertion and deletion.

## 3. Results

### 3.1. Simulation Studies

In the simulation study, we investigate the impact of over-selection on prediction accuracy and compare the variable selection and prediction performance of our repeated sieving method with LASSO and EN. LASSO and EN do not control the number of selection, while our repeated sieving method, nested with R-SVS, controls the number of selections by setting various alpha values for insertion and deletion. We denote $\left(\alpha_{1},\alpha_{2}\right)$ as the alpha values for insertion and deletion for R-SVS within blocks and $\left(\alpha_{3},\alpha_{4}\right)$ as those for the final variable selection.

We generate n=400 samples of m=10,000 features using a multivariate Gaussian distribution with mean 0 and variances 1. The random vector of features is generated to consist of 500 independent blocks with block size 20. Within each block, the multivariate Gaussian distribution has a compound symmetry structure with a common correlation coefficient ρ=0.2. We assume that $m_{1}=6$ predictors out of the m=10,000 candidate predictors are truly associated with the outcome, and every two of them belong to the same blocks. Thus, the $m_{1}=6$ true predictors belong to three different blocks.

At first, we consider a binary outcome case. For subject i(=1,…,n) and the true predictors ${\tilde{z}}_{i}={\left(z_{\tilde{1}i},\ldots ,z_{{\tilde{m}}_{1}i}\right)}^{T}$, the outcome $y_{i}$ is generated from a Bernoulli distribution with the success probability fitted using the logistic regression model

$$p_{i}=P\left(y_{i}=1\right)=\frac{\exp(\beta_{0}+\beta^{T}{\tilde{z}}_{i})}{1+\exp(\beta_{0}+\beta^{T}{\tilde{z}}_{i})},$$

where $\beta ={\left(\beta_{\tilde{1}},\ldots ,\beta_{{\tilde{m}}_{1}}\right)}^{T}$ is the vector of regression coefficients corresponding to the true predictors ${\tilde{z}}_{i}$. We set the values of regression coefficients as $\beta_{\tilde{l}}={\left(-1\right)}^{l+1}*2,l=0,1,\ldots ,m_{1}$, and $\beta_{\tilde{0}}=0$ is the intercept term. We use 50–50 hold-out for a training set of size $n_{1}=200$ and a validation set of size $n_{2}=200$.

By performing more permutations, the chance of jointly selecting true covariates from the same block becomes higher. We prove this idea by comparing the results of our repeated sieving method with P=10 and 100 permutations. For each permuted data set, we partition the covariates into blocks with $m_{0}=50$ covariates each, for which L-SVS can be easily carried out. We apply L-SVS to each block of the training set, and, after *P* permutations, the candidate covariates for the final model are the union of all sieved (or selected) covariates from all permutations. The alpha values $\left(\alpha_{1},\alpha_{2}\right)=\left(0.01,0.02\right)$ are found to select around 200 sieved candidate covariates. The L-SVS applied to the set of these candidate covariates uses $\left(\alpha_{3},\alpha_{4}\right)=\left(0.0025,0.005\right)$ to fit the final prediction model.

For the fitted final prediction model, we count the total number of covariates included in the model, called total selection, and the number of true covariates among them, called true selection. Let *Z* denote the vector of covariates selected by the final model and $\hat{\beta }$ the corresponding regression estimates. Then, we calculate the AUC value using the fitted risk score $r_{i}={\hat{\beta }}^{T}Z_{i}$ and outcome $y_{i}$ from the training set and the validation set. We repeat this simulation N=100 times, and calculate the mean total selection and mean true selection from the training sets, together with the mean AUC from the training and validation sets. AUC for the training set measures how well the final prediction model fits the training data, but it does not measure the real prediction accuracy because the fitted model tends to fit the training set better by including more predictors [20]. This is called an over-fitting issue. The AUC value from an independent validation set really measures the prediction accuracy, resolving the over-fitting issue.

The simulation results are summarized in Table 1. The $L_{1}$-norm penalty of LASSO is stricter than the $L_{1}$- and $L_{2}$-norm combination penalty of EN, so that LASSO has a smaller mean total selection than EN. EN also has a little more true selection than LASSO, probably because of the larger total selection. The results also show that LASSO and EN select a large number of features, while the repeated sieving methods only select fewer than 10 features in total. The true selections of the two ML methods are slightly larger than that of repeated sieving methods, but considering their big total selections, this difference in true selection is negligible.

By selecting a much larger number of covariates, the two ML methods have a slightly better fitting of the training sets than our repeated sieving. However, from the average AUCs for validation sets, we find that our repeated sieving method has better prediction accuracy than LASSO and EN. We have these results because the ML methods have large proportions of false selections that act like error terms in the fitted prediction models and, hence, lower the prediction accuracy.

Overall, the repeated sieving method has better prediction accuracy, even with a much smaller number of total selections than LASSO and EN. On the other hand, for our repeated sieving method, by performing more permutations (i.e., P=10 vs. P=100), both total and true selections increase, but the increase in true selection is larger. Hence, repeated sieving with P=100 permutations has a slightly higher AUC from validation sets.

For the survival outcome case, the covariates are generated in the same way as in the binary outcome case. For subject i(=1,…,n) with true predictors $z_{i}={\left(z_{\tilde{1}i},\ldots ,z_{{\tilde{m}}_{1}i}\right)}^{T}$, the hazard rate of the survival distribution is given by

$$h_{i}\left(t\right)=h_{0}\left(t\right)e^{\beta^{T}z_{i}}$$

where $\beta ={\left(\beta_{\tilde{1}},\ldots ,\beta_{{\tilde{m}}_{1}}\right)}^{T}$ is the vector of regression coefficients and $h_{0}\left(t\right)$ is an unknown baseline hazard function. The $m_{1}=6$ true features are drawn in the same way as in the binary outcome case, and we set $\beta_{\tilde{l}}={\left(-1\right)}^{l+1}*0.7$ for $l=1,2,\ldots ,m_{1}$ with baseline hazard rate $h_{0}=0.1$. We consider either 30% or 10% censoring proportion. For the 30% censoring case, censoring times are generated from a uniform distribution U(0,a). Fixing the accrual period *a*, the censoring times for 10% censoring are generated from another uniform distribution U(a,a+b) with an additional follow-up period *b*. We apply 50–50 hold-out for splitting a data set of size n=400.

The process of repeated sieving for survival outcome is the same as that for binary outcome, except the nested L-SVS is replaced with C-SVS. We compare the performance of LASSO, EN, and the repeated sieving using C-SVS with P=10 or 100 permutations. For the repeated sieving, we use $\left(\alpha_{1},\alpha_{2}\right)=\left(0.01,0.02\right)$ to select fewer than 500 candidate features from the permuted features and $\left(\alpha_{3},\alpha_{4}\right)=\left(0.0005,0.001\right)$ for the final prediction model by applying C-SVS to the set of candidate features. We count the total selection and the true selection by the final prediction model fitted from the training set.

Let *Z* denote the selected predictors by the final model and $\hat{\beta }$ their corresponding coefficients; then, we fit a univariate Cox regression model with the survival outcome $\left(y_{i},\delta_{i}\right)$ on the covariate $r_{i}={\hat{\beta }}^{T}Z_{i}$ using the training or the validation set. The *p*-value of the single covariate is calculated and $-\log_{10}$*p*-value is used to measure the prediction accuracy. We also estimate the association between the risk score $r_{i}$ and the outcome $\left(y_{i},\delta_{i}\right)$ using Harrell’s C-index for the training and validation sets. Large $-\log_{10}$*p*-value and C-index for the training set mean that the final prediction model fits the training set well, and those for the validation set indicate that the fitted model has a good prediction accuracy. Through N=100 simulations, the mean total and true selections are calculated from the training sets, and the mean $-\log_{10}$*p*-value and C-index are calculated from both training and validation sets.

The simulation results for the survival outcome case are shown in Table 2. With 10% censoring, all the methods have more true selections and higher prediction accuracy in terms of $-\log_{10}$*p*-value and C-index for validation sets than with 30% censoring. As in the binary outcome case, LASSO and EN have much larger total selections than our repeated sieving method. With 30% censoring, the mean true selections of LASSO and EN are slightly larger than that of our repeated sieving method. With 10% censoring, however, the mean true selection of the repeated sieving with P=100 permutations is higher than LASSO and EN. With much smaller total selection and high true selection, our repeated sieving method has higher prediction accuracy than LASSO and EN overall. The results also show that the repeated sieving method with P=100 permutations has higher true selection and higher prediction accuracy in terms of $-\log_{10}$*p*-value and C-index from validation sets.

### 3.2. Real Data Examples

Wang et al. [21] published a data set from the tumour bank at the Erasmus Medical Center (Rotterdam, Netherlands) collected from frozen tumour samples of patients with lymph-node-negative breast cancer. The patients received treatment during 1980–1995, but they did not receive systemic neoadjuvant or adjuvant therapy. This human recurrence breast cancer microarray data set can also be retrieved from the Gene Expression Omnibus database. This data set contains n=286 samples and the expression of m=22,283 transcripts from total RNA of the samples. Estrogen receptor (ER) status denotes the cell type where ER+ means normal cells and ER- stands for abnormal cells. The relapse-free survival (RFS) rates of the patients are also included in the data set.

**Figure 1:**
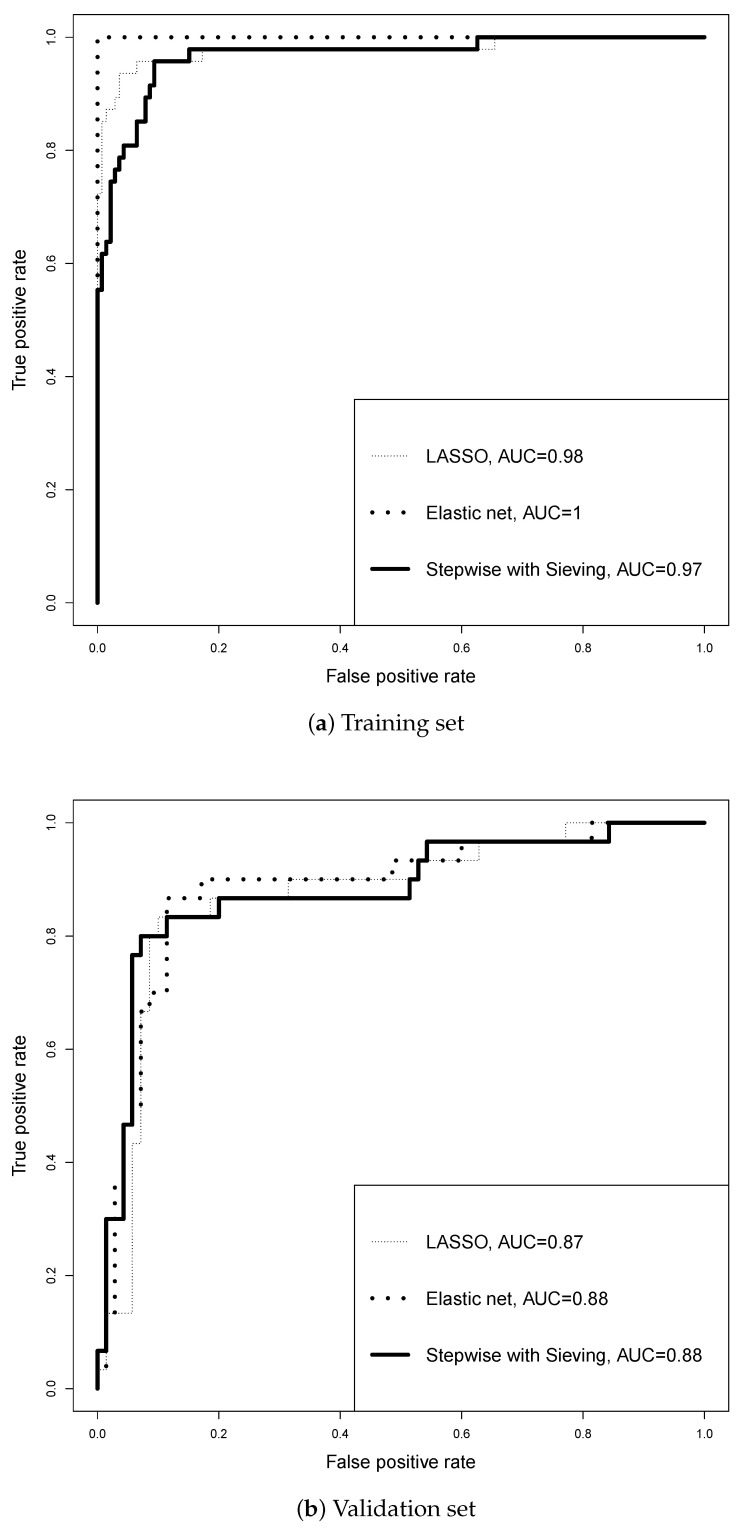
ROC curves from the prediction of ER status using different methods for Wang et al.’s [21] data.

For the binary outcome of ER status, we randomly select $n_{2}=100$ samples for validation and the remaining $n_{1}=186$ samples are used to train the model. LASSO, EN, and repeated sieving with P=100 permutations are applied to fit a prediction model. For the repeated sieving method, we partition the features into blocks of size $m_{0}=50$ and alpha values $\left(\alpha_{1},\alpha_{2},\alpha_{3},\alpha_{4}\right)=\left(0.000001,0.000002,0.0025,0.005\right)$. Figure 1 shows the ROC curves of the fitted models for the training and validation sets. All three models have large AUCs for the training set because of over-fitting. But, for the validation set, EN and repeated sieving have slightly larger AUC than LASSO. More analysis results are shown in Table 3. The repeated sieving method selects only two features (that are also selected by LASSO and EN), compared with 22 for LASSO and 520 for EN, but it has the same prediction accuracy as or slightly higher prediction accuracy than the ML methods.

From these results, we find that LASSO and EN select too many features and many of them are false selections. For the binary outcome of ER-status, we apply L-SVS to the set of features included in the final prediction models by LASSO and EN, called LASSO-set and EN-set, respectively, using $\alpha_{1}=0.0025$ for insertion and $\alpha_{2}=0.005$ for deletion. Since repeated sieving selects only two features, we do not have to further reduce the number of features. Table 4 reports the results, comparing the performance of the final prediction models fitted by L-SVS from LASSO-set and EN-set. Note that the fitted model, obtained by applying L-SVS to the LASSO-set, selects exactly the same two features as repeated sieving, so that these two methods have the same final prediction models and identical prediction performance. However, the final model of L-SVS applied to EN-set selects three features, among which only one is commonly selected by the other two methods. In spite of one more selection than the other two methods, the final model by L-SVS applied to EN-set has slightly lower prediction accuracy in terms of AUC from the validation set. Combined with L-SVS, LASSO and EN have much fewer selections (2 vs. 22 and 3 vs. 520, respectively) while maintaining similar prediction accuracy as our repeated sieving. This demonstrates the issues associated with over-selection. We apply multivariate regression to the models from Table 4 and summarize the coefficients and significance of the selected covariates in Table 5.

For the time-to-event outcome of RFS, we use the same training and validation sets as in the ER-status analysis. We apply LASSO, EN, and repeated sieving with P=100 permutations, block size for sieving $m_{0}=50$, and alpha values $\left(\alpha_{1},\alpha_{2},\alpha_{3},\alpha_{4}\right)=(0.0001,$ 0.0002, 0.0005, 0.001) to the training set. The analysis results are summarized in Table 6. As in the analysis of ER status, repeated sieving selects much fewer features than LASSO and EN. Due to the over-selection, LASSO and EN fit the training data better in terms of larger $-\log_{10}$*p*-value and C-index, but repeated sieving has the highest prediction accuracy in terms of its highest $-\log_{10}$*p*-value and C-index for validation set. We find that five out of the nine features selected by repeated sieving are also selected by both LASSO and EN.

Since LASSO and EN select too many features, we apply C-SVS to the sets of features selected by LASSO and EN to further reduce the dimension. Table 7 reports the performance of the final models and the list of features included in the final model. The three methods select a similar number of features and 219312_s_at is selected by all three models. By applying C-SVS to LASSO-set and EN-set, we achieve significant dimension reduction (to 8 from 54 and to 10 from 174). By comparing the $-\log_{10}$*p*-value and C-index for the validation set between Table 5 and Table 6, we find that EN followed by C-SVS (Table 6) improves the prediction accuracy drastically from EN alone (Table 5), although LASSO followed by C-SVS does not improve the prediction accuracy from LASSO alone at all. Table 8 summarizes the coefficient size and significance of the covariates from the fitted models in Table 7.

## 4. Discussion

ML methods such as LASSO and EN have been widely used to develop prediction models with high-dimensional data, and the recent recognition of ML methods promotes collective applications and investment. This feat stimulates the development of clinical data analysis but also induces potential overuse risk since the over-selection and unfavorable prediction accuracy issues have been identified and reviewed by many studies. Liu et al. [3] provide an alternative approach, which combines standard regression methods with stepwise variable selection, called R-SVS here. We extend their method to prediction model building with very high-dimensional data. Both simulations and real data examples show that the ML methods over-select features and lose prediction power due to over-selecting features. However, our repeated sieving method has much fewer selections but higher selection precision and better prediction accuracy compared with LASSO and EN.

There has been some recent research on variants of LASSO trying to resolve the over-selection problem [22,23], but we only consider the traditional LASSO in this paper and it may be our future topic to compare the performance between these modified ML methods with our repeated sieving method. We find that the repeated sieving method produces precise prediction models with efficient computing, but without the complicated modifications as in these ML models.

SAS has a program that performs stepwise variable selection that uses information criteria such as AIC, BIC, or R-square. Like LASSO and EN, the stepwise selection procedures using this information cannot control the amount of selection. We extend the R program for R-SVS developed by Liu et al. [3] and propose the repeated sieving approach to handle high-dimensional data. Even though it is known that traditional regression is not appropriate for high-dimensional data analysis, our repeated sieving method resolves this problem by partitioning the high-dimensional features into low-dimensional blocks for which R-SVS can be conducted efficiently. Note that the repeated sieving method is more applicable, especially when the number of truly significant predictors is much smaller than the sample size. Our repeated sieving method can be applied to any type of outcomes as far as regression methods exist. Further, it is composed of standard statistical methods, so that researchers without deep knowledge of bioinformatics theory can still use it for analyzing high-dimensional data. Our software is available at the link https://github.com/tsluffy1994/Repeated-Sieving-for-Prediction-Model-Building-with-High-Dimensional-Data in GitHub (accessed on 18 June 2024).

In SVS, $\left(\alpha_{1},\alpha_{2},\alpha_{3},\alpha_{4}\right)$ can be selected so that the final model includes a reasonable number of features. We may try different alpha values so that the *p*-value from validation set is minimized. The repeated sieving method in this paper has a common goal with the SVS by Liu et al. [3], i.e., prediction model building from high-dimensional data, but the ideal dimensionality of data is different between the two methods. SVS works well for a data set with up to *m* = 1000 features, but repeated sieving is more appropriate for data with a larger number of features, such as m>10,000. If the final prediction model includes *K* features, the number of regression models to be fitted is on the order $O(m^{K+1})$ for SVS, while it is on the order O(m). If *m* is large, e.g., in the millions, this difference will be huge, so that SVS will require excessive computing time, whereas the sieving method can be used almost limitlessly in the dimension of data.

The repeated sieving method takes a little more computing time to train models than LASSO and EN, since they select variables in different ways. Repeated sieving, which is nested with stepwise selection, spends most of the running time performing hypothesis testing and calculating the *p*-values of covariates for variable selection based on statistical significance. However, LASSO and EN just estimate regression coefficients to select covariates based on their sizes. Another process that makes repeated sieving require a longer computing time is permutations. We find that repeated sieving with 100 permutations has slightly higher prediction performance than with 10 permutations. The computing time of LASSO, EN, and repeated sieving for real data analysis are 8.76 s, 291.6 s, and 13,237.1 s for binary outcome, and 79.46 s, 887.62 s, and 13,204.85 s for survival outcome. It is expected that repeated sieving takes the most time because it conducts P=100 permutations, and the running time can be further reduced with parallel computing power.

## Figures and Tables

**Table 1:** Binary outcome case: Simulation results of the three prediction methods with m=10,000 total features and $m_{1}=6$ true features. Repeated sieving method uses $\left(\alpha_{1},\alpha_{2}\right)=\left(0.01,0.02\right)$, $\left(\alpha_{3},\alpha_{4}\right)=\left(0.0025,0.005\right)$, and P(=10 or 100) permutations.

			Repeated Sieving	
	**LASSO**	**EN**	P=10	P=100
Total Selections	67.05	94.6	8.61	8.77
True Selections	5.71	5.77	5.31	5.66
AUC-Training	1.00	1.00	0.98	0.99
AUC-Validation	0.81	0.79	0.87	0.89

**Table 2:** Survival outcome case: Simulation results of the three prediction methods with m=10,000 total features and $m_{1}=6$ true features. Repeated sieving method uses $\left(\alpha_{1},\alpha_{2}\right)=\left(0.01,0.02\right)$, $\left(\alpha_{3},\alpha_{4}\right)=\left(0.0005,0.001\right)$, and P(=10 or 100) permutations.

			Repeated Sieving	
	**LASSO**	**EN**	P=10	P=100
	(i) 30% Censoring			
Total Selections	33.93	40.4	8.26	9.63
True Selections	5.58	5.63	5.17	5.54
$-\log_{10}p\text{-}\mathrm{value}$ (training)	41.10	42.74	34.67	37.05
$-\log_{10}p\text{-}\mathrm{value}$ (validation)	19.18	18.55	21.02	22.65
C-index (training)	0.84	0.86	0.82	0.83
C-index (validation)	0.72	0.72	0.73	0.74
	(ii) 10% Censoring			
Total Selections	35.29	36.71	8.25	9.4
True Selections	5.68	5.77	5.69	5.83
$-\log_{10}p\text{-}\mathrm{value}$ (training)	47.22	47.33	40.58	42.37
$-\log_{10}p\text{-}\mathrm{value}$ (validation)	26.45	25.83	28.97	29.72
C-index (training)	0.83	0.84	0.81	0.82
C-index (validation)	0.74	0.74	0.75	0.76

**Table 3:** Analysis results of Wang et al.’s [21] data on ER status.

Method	# Selected Features	AUC-Training	AUC-Validation
LASSO	22	0.98	0.87
Elastic Net	520	1.00	0.88
Repeated Sieving	2	0.97	0.88

**Table 4:** Analysis results of Wang et al.’s [21] data on ER status when applying L-SVS to the sets of features selected by LASSO and EN.

Method	# Selected Features	AUC-Training	AUC-Validation
LASSO-stepwise	2	0.97	0.88
Elastic Net-stepwise	3	0.98	0.86
Repeated Sieving	2	0.97	0.88
	Selected Features		
LASSO-stepwise	209604_s_at, 218146_at		
Elastic Net-stepwise	209604_s_at, 207754_at, 204495_s_at		
Repeated Sieving	209604_s_at, 218146_at		

**Table 5:** Multivariate logistic regression on ER status using the training set of Wang et al.’s [21] data.

LASSO-Stepwise			Elastic Net-Stepwise		
**Feature**	**Coef.**	***p*** **-Value**	**Feature**	**Coef.**	***p*** **-Value**
209604_s_at	$-2.97\times 10^{-4}$	$5.74\times 10^{-9}$	209604_s_at	$-5.62\times 10^{-4}$	$1.49\times 10^{-6}$
218146_at	$-2.68\times 10^{-3}$	$5.98\times 10^{-4}$	207754_at	$-4.98\times 10^{-2}$	$7.36\times 10^{-5}$
			204495_s_at	$1.38\times 10^{-2}$	$1.46\times 10^{-4}$
**Repeated Sieving**					
**Feature**		**Coef.**		***p*** **-Value**	
209604_s_at		$-2.97\times 10^{-4}$		$5.74\times 10^{-9}$	
218146_at		$-2.68\times 10^{-3}$		$5.98\times 10^{-4}$	

**Table 6:** Analysis results of Wang et al.’s [21] data on RFS.

	# Selected	$-\log_{10}$*p*-Value		C-Index	
**Method**	**Features**	**Training**	**Validation**	**Training**	**Validation**
LASSO	54	30.71	0.80	0.94	0.62
Elastic Net	174	29.60	0.49	0.95	0.58
Repeated Sieving	9	26.43	2.65	0.85	0.64

**Table 7:** Analysis results of Wang et al.’s [21] data on RFS when applying C-SVS to the sets of features selected by LASSO and EN.

	# Selected	$-\log_{10}$*p*-Value		C-Index	
**Method**	**Features**	**Training**	**Validation**	**Training**	**Validation**
LASSO-stepwise	8	24.45	0.23	0.84	0.54
Elastic Net-stepwise	10	26.65	3.78	0.87	0.68
Repeated Sieving	9	26.43	2.65	0.85	0.64
	Selected Features				
LASSO-stepwise	219312_s_at, 219408_at, 218270_at, 212431_at, 212900_at,				
	207763_at, 201598_s_at, 212898_at				
Elastic Net-stepwise	219312_s_at, 203218_at, 216822_x_at, 219478_at, 212990_at,				
	205239_at, 219116_s_at, 214592_s_at, 212431_at, 217840_at				
Repeated Sieving	219312_s_at, 203218_at, 216822_x_at, 219478_at, 209524_at,				
	211004_s_at, 206012_at, 204991_s_at, 205551_at				

**Table 8:** Multivariate Cox regression on RFS using the training set of Wang et al.’s [21] data.

LASSO-Stepwise			Elastic Net-Stepwise		
**Feature**	**Coef.**	***p*** **-Value**	**Feature**	**Coef.**	***p*** **-Value**
219312_s_at	$5.55\times 10^{-3}$	$1.31\times 10^{-6}$	219312_s_at	$6.66\times 10^{-3}$	$1.51\times 10^{-8}$
219408_at	$1.12\times 10^{-2}$	$1.91\times 10^{-14}$	203218_at	$1.11\times 10^{-3}$	$9.46\times 10^{-9}$
218270_at	$-1.30\times 10^{-3}$	$6.83\times 10^{-7}$	216822_x_at	$1.76\times 10^{-2}$	$6.49\times 10^{-11}$
212431_at	$-4.40\times 10^{-3}$	$3.14\times 10^{-6}$	219478_at	$4.07\times 10^{-3}$	$2.20\times 10^{-8}$
212900_at	$2.12\times 10^{-3}$	$3.55\times 10^{-5}$	212990_at	$6.45\times 10^{-3}$	$2.64\times 10^{-8}$
207763_at	$2.83\times 10^{-2}$	$8.72\times 10^{-8}$	205239_at	$2.89\times 10^{-4}$	$7.74\times 10^{-4}$
201598_s_at	$1.80\times 10^{-3}$	$1.86\times 10^{-6}$	219116_s_at	$3.63\times 10^{-3}$	$1.21\times 10^{-9}$
212898_at	$1.21\times 10^{-3}$	$1.99\times 10^{-4}$	214592_s_at	$1.61\times 10^{-2}$	$2.65\times 10^{-5}$
			212431_at	$-5.37\times 10^{-3}$	$1.11\times 10^{-6}$
			217840_at	$1.27\times 10^{-3}$	$8.62\times 10^{-5}$
**Repeated Sieving**					
**Feature**		**Coef.**		***p*** **-Value**	
219312_s_at		$6.07\times 10^{-3}$		$4.18\times 10^{-7}$	
203218_at		$1.08\times 10^{-3}$		$1.01\times 10^{-9}$	
216822_x_at		$1.35\times 10^{-2}$		$1.10\times 10^{-9}$	
219478_at		$4.98\times 10^{-3}$		$9.91\times 10^{-12}$	
209524_at		$7.54\times 10^{-4}$		$8.63\times 10^{-6}$	
211004_s_at		$-6.15\times 10^{-3}$		$5.82\times 10^{-5}$	
206012_at		$1.06\times 10^{-3}$		$2.06\times 10^{-5}$	
204991_s_at		$-1.73\times 10^{-2}$		$1.50\times 10^{-5}$	
205551_at		$1.97\times 10^{-3}$		$7.43\times 10^{-5}$	

## Data Availability

No new data were created or analyzed in this study. Data sharing is not applicable to this article.
